# Neuromyelitis Optica in Child: Diagnostic and Therapeutic Challenges

**DOI:** 10.1155/2013/124929

**Published:** 2013-12-11

**Authors:** Karolina Dembinski, Maria Gieron-Korthals, Carlos R. Martinez, Lisa Rodriguez

**Affiliations:** ^1^Community Health Centers, Lompoc, CA 93436, USA; ^2^Division of Child Neurology, Department of Pediatrics, University of South Florida, Morsani College of Medicine, Tampa, FL 33606, USA; ^3^Division of Neuroradiology, Department of Radiology, University of South Florida, Morsani College of Medicine, Tampa, FL 33606, USA; ^4^Department of Pediatrics, University of South Florida, Morsani College of Medicine, Tampa, FL 33606, USA

## Abstract

Neuromyelitis optica (NMO) is a rare syndrome of severe inflammatory demyelination of the central nervous system, causing attacks of optic neuritis and transverse myelitis. Although uncommon, attention should be given to the proper identification and management of the affected patients. We present a case of a 13-year-old girl with severe neuromyelitis optica. The patient's initial presentation consisted of encephalopathy and optic neuritis. Approximately 2 months later, coinciding with the weaning of steroid treatment, she presented with ascending paralysis and respiratory failure. She was seropositive for NMO-IgG. Treatment included intravenous immune globulin, steroids, plasmapheresis, and rituximab and was complemented with proper nutrition, vitamins, minerals, and intense rehabilitation. Two years after the initial presentation and one short relapse, the patient has made a remarkable recovery without neurologic deficit. This report underscores the difficulty in making the initial diagnosis, choosing the best treatment, and the need for more streamlined pediatric guidelines for diagnosis, treatment, and prevention of relapses of pediatric NMO.

## 1. Introduction

Neuromyelitis optica (NMO), otherwise known as Devic Disease, is an uncommon clinical syndrome of central nervous system inflammatory demyelination, comprising of optic neuritis and transverse myelitis. First accounts to this disorder date back to the 1870s [1]. Although once believed a variant of other disorders like multiple sclerosis, acute demyelinating encephalomyelitis (ADEM), systemic lupus erythematosus, Sjögren's syndrome, and connective tissue disorders, as well as association with viral and bacterial infections [2], specific diagnostic criteria have now been proposed for NMO, making it a clinically distinct syndrome [3]. Early recognition of NMO is important for specific management options and proper patient and family counseling. If undiagnosed, NMO may lead to unnecessary investigations and treatments, recurrent and prolonged hospital course, significant morbidity, and even death. We report this case to show a protracted course of the disease, diagnostic and treatment difficulties, and to raise awareness about this rare disorder among pediatricians who are usually first involved in the care of these children.

## 2. Case Presentation

### 2.1. First Admission

A 13-year-old African American girl presented to the hospital with a 6-day history of fatigue, weakness, upper back and shoulder pain, subjective fever, decreased appetite, and weight loss. She was previously healthy. On examination, she was encephalopathic with waxing and waning mental status, flat affect, ophthalmoparesis, with decreased visual acuity, and color agnosia. During hospitalization, the patient's symptoms worsened with episodes of vomiting, periods of agitation, and at times unresponsiveness to environmental stimuli.

**Figure 1:**
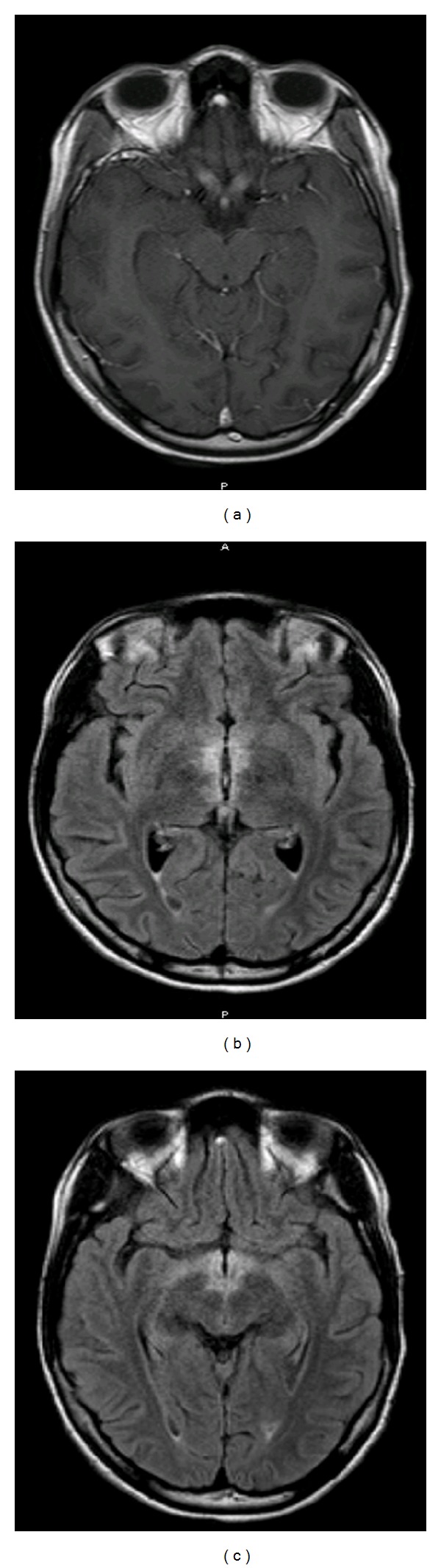
MRI of the brain: (a) axial T1W postcontrast: enhancement of bilateral optic nerves extending to the optic chiasm. (b) and (c): T2W FLAIR: increased signal in a symmetric fashion involving the hypothalami, mammillary bodies, periaqueductal gray matter, and posterior aspect of pons and medulla.

The initial brain MRI showed increased signal in the hypothalami, mammillary bodies, periaqueductal gray matter, and posterior aspect of pons and medulla, raising a question about Wernicke's encephalopathy. Subsequent MRIs showed progression of abnormalities with new enhancement of bilateral optic nerves extending to the optic chiasm, pontine tegmentum bilaterally, and gradual resolution of earlier appeared changes (Figure 1).

EEG showed bilateral slowing consistent with encephalopathy.

The cerebrospinal fluid (CSF) examination showed 157 WBC/mm^3^ with 95% lymphocytes. Aquaporin-4 receptor antibody in serum was negative.

Patient was treated with intravenous immune globulin (IVIG), 2 doses (1 g/kg) over a 48-hour period and thiamine for suspected Wernicke's encephalopathy based on the presence of encephalopathy clinically and suggestive MRI findings. Subsequent thiamine level was normal and she had not improved with thiamine treatment. Secondary to worsening clinical condition, patient was started on a 5-day course of intravenous methylprednisolone (30 mg/kg for 3 days, then 15 mg/kg for 2 days), followed by a 5-week oral prednisone taper. The patient responded to steroid treatment with significant improvement in her mental status, vision, and musculoskeletal exam. She was also diagnosed with latent tuberculosis based on positive QuantiFERON-TB Gold test (with negative PPD and chest X-ray) and started on a 9-month treatment regimen with isoniazid and pyridoxine. It was undetermined whether latent tuberculosis was related to her illness. After a 2-month inpatient stay, including 1 month of rehabilitation, she was discharged home on prednisone taper, multivitamins, and minerals with continued outpatient rehabilitation.

### 2.2. Second Admission

Approximately 1.5 months later, patient presented to the emergency room with a two-week history of progressive paresthesias, leg weakness, and bladder and bowel dysfunction. Patient decompensated quickly and progressed to quadriplegia, respiratory failure, and altered mental status. She required intubation and mechanical ventilation for 8 days in the intensive care unit.

**Figure 2:**
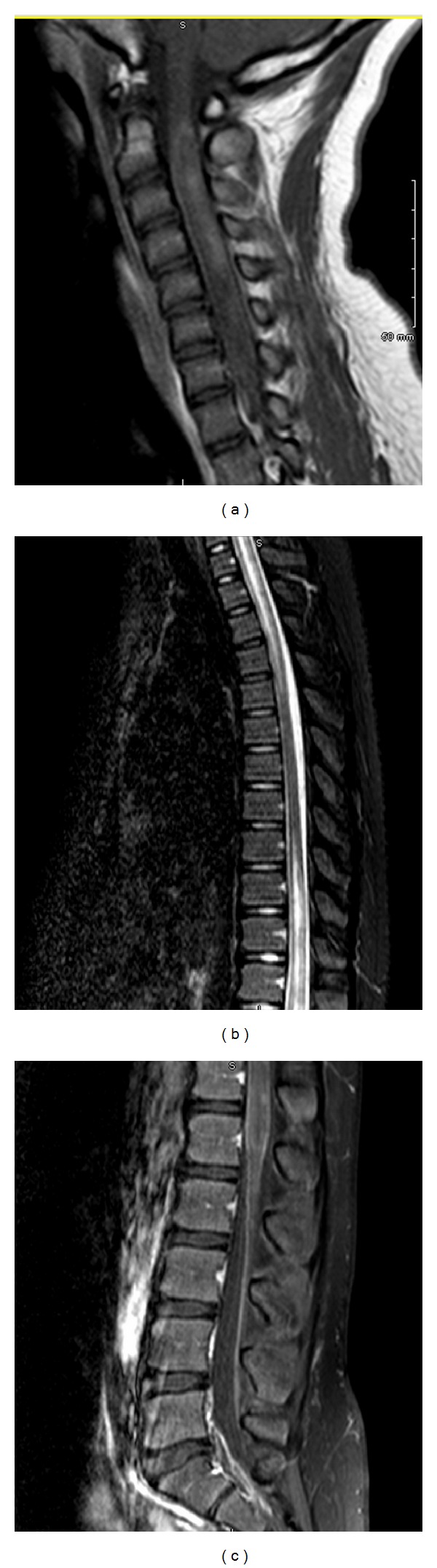
MRI of the cervical spine: (a) T1W postcontrast shows increased cord signal with an enlarged, edematous central cord. MRI of the thoracic spine: (b) T2W STIR shows abnormal T2 signal within the cord. MRI of the lumbar spine: (c) T1W postcontrast with fat suppression shows abnormal enhancement in the cord.

MRI of the spine showed increased signal throughout most of the cervical and thoracic spine, with central cord enlargement, and heterogeneous and patchy areas of central and peripheral enhancement (Figures 2(a)–2(c)). MRI of the brain was normal this time. Abnormal lab findings were: NMO-IgG antibodies positive in CSF and serum; positive EBV by PCR (119 copies/mL in CSF and 136 copies/mL in serum), EBV IgG positive, EBV IgM negative; low copper level of 60 (75–187 mcg/dL), and positive ANA screen.

At this point, the patient was diagnosed with seropositive neuromyelitis optica.

Treatments included IV methylprednisolone at 1 to 2 grams daily for 7 days, 1 dose of IVIG at 1 g/kg, plasmapheresis on alternate days for 7 days, and IV cupric chloride (1.2 mg daily for approximately 2 weeks) as a copper supplement, as well as a multivitamin with mineral daily. She also received 4 treatments of rituximab at 375 mg/m^2^, given every other week, outpatient.

After about 2 months of inpatient care, she was transferred to the inpatient rehabilitation service for therapies. At the time of discharge, 3.5 months after the second admission, her visual acuity and color agnosia had improved, she had regained partial use of her upper extremities and some improvement in sensation of lower extremities but still with paraplegia, requiring manual wheelchair for mobility and motorized wheelchair only in school. She also had some awareness of her bowel/bladder function, although remained incontinent. Medications on discharge included slow prednisone taper, gabapentin for pain, rituximab weekly to complete 4 doses, and multivitamin with minerals. She continued outpatient rehabilitation. The MRIs 10 months later showed normal brain and cervical cord spine while thoracic cord showed T2 signal abnormalities with minimal residual patchy enhancement in mid and lower thoracic cord.

### 2.3. Third Admission

She was in outpatient rehab for 6.5 months and at the time of discharge walked independently without any assistance. She continued doing well for another 5 months except for some episodic urinary incontinence and then had a relapse with mild right-sided weakness and numbness. MRI brain was normal, cervical MRI was markedly abnormal with new cord enlargement at C2 and C3 with associated abnormal enhancement. Thoracic cord remained unchanged from examination 6 months earlier. Repeated CSF examination was normal except for elevated myelin basic protein to 15.3 *μ*L (nl 0.0–4.0 *μ*L). She was treated with 3 doses of methylprednisolone, followed by 4 doses of rituximab at 375 mg/m^2^ given weekly. She made a remarkable recovery and, 5 months after the relapse, she is neurologically completely normal, does well in school, and wants to be a pediatric neurologist. She received the first preventive treatment 7 months after the last one while her total B-cell count was low: CD19 absolute count < 20 cells/*μ*L (nl 130–180) or 1% of normal.

## 3. Discussion

Proposed diagnostic criteria for NMO in children require the following features: optic neuritis, myelitis, and one of the following: MRI evidence of a contiguous spinal cord lesion three or more segments in length or seropositivity for NMO-IgG. NMO-IgG autoantibody that selectively binds to the aquaporin-4 (AQP4) water channel is thought to be a specific marker for NMO [4]. It is present in over 70 percent of those with NMO and in a smaller percentage of other demyelinating CNS disorders, including multiple sclerosis [5]. In patients who meet these criteria, some other CNS involvement beyond the optic nerves and spinal cord is compatible with NMO [4].

The clinical course of NMO is variable. It may occur as a monophasic illness that is either fulminant and fatal or associated with varying degrees of recovery. Pediatric cases typically have a monophasic course and many have complete neurological recovery [2, 6]. Polyphasic courses characterized by relapses and remissions also occur. Our patient was diagnosed with seropositive NMO upon second presentation with myelitis. It is likely that our patient has had biphasic presentation with the first phase being encephalopathy and optic neuritis and the second phase with myelitis and encephalopathy.

In the pediatric population, NMO is frequently preceded by infection. Positive CSF and blood for EBV in our patient was suspicious of being a causative factor.

There are no specific guidelines for NMO treatment. At present, the mainstay of therapy is the treatment of acute attacks, prevention of medical complications, and rehabilitation. Specific medications that have been used include: methylprednisolone with prednisone taper, immunosuppressants such as azathioprine [7, 8], cyclophosphamide [8, 9], and rituximab [10, 11], and plasmapheresis [12–15]. Rituximab is a murine/human monoclonal antibody directed against CD20 that induces B-cell depletion when administered in vivo. It is approved by the FDA for the treatment of CD20+ lymphomas and rheumatoid arthritis. Its selective depletion of the humoral component of the immune system makes it an attractive and more effective approach for treating NMO [11]. The rapid succession of various treatments in our patient with rituximab being last, and supplement with cupric chloride for low copper level, makes it difficult to determine which one combination was the best one and decreased her disability. She responded very well to rituximab during a short-lived relapse 15 months after the diagnosis of NMO was made. We choose rituximab for the prevention of relapses in this patient. Although at present she is completely normal, this outcome did not come easily nor timely and her future remains uncertain.

## 4. Conclusion

This case report should serve as an example of the difficulties pediatricians and neurologists encounter caring for children with NMO. Furthermore, it calls for establishing diagnostic criteria and evidence-based guidelines for the treatment and relapse prevention of pediatric NMO.
